# Estimating malaria transmission from humans to mosquitoes in a noisy landscape

**DOI:** 10.1098/rsif.2015.0478

**Published:** 2015-10-06

**Authors:** Robert C. Reiner, Carlos Guerra, Martin J. Donnelly, Teun Bousema, Chris Drakeley, David L. Smith

**Affiliations:** 1 Fogarty International Center, National Institutes of Health, Bethesda, MD, USA; 2 Department of Entomology, University of California, Davis, CA, USA; 3 Department of Epidemiology and Biostatistics, Indiana University, Bloomington, IN, USA; 4 Center for Disease Dynamics, Economics and Policy, Washington, DC, USA; 5 Department of Vector Biology, Liverpool School of Tropical Medicine, Pembroke Place, Liverpool, UK; 6 Malaria Programme, Wellcome Trust Sanger Institute, Cambridge, UK; 7 Department of Immunology and Infection, London School of Hygiene and Tropical Medicine, London, UK; 8 Department of Medical Microbiology, Radboud University Nijmegen Medical Centre, Nijmegen, The Netherlands; 9 Sanaria Institute for Global Health and Tropical Medicine, Rockville, MD, USA; 10 Department of Zoology, University of Oxford, Oxford, UK; 11 Institute for Health Metrics and Evaluation, University of Washington, Seattle, WA, USA

**Keywords:** disease ecology, infectious disease dynamics, mosquito-borne pathogen, non-Markovian dynamics

## Abstract

A basic quantitative understanding of malaria transmission requires measuring the probability a mosquito becomes infected after feeding on a human. Parasite prevalence in mosquitoes is highly age-dependent, and the unknown age-structure of fluctuating mosquito populations impedes estimation. Here, we simulate mosquito infection dynamics, where mosquito recruitment is modelled seasonally with fractional Brownian noise, and we develop methods for estimating mosquito infection rates. We find that noise introduces bias, but the magnitude of the bias depends on the ‘colour' of the noise. Some of these problems can be overcome by increasing the sampling frequency, but estimates of transmission rates (and estimated reductions in transmission) are most accurate and precise if they combine parity, oocyst rates and sporozoite rates. These studies provide a basis for evaluating the adequacy of various entomological sampling procedures for measuring malaria parasite transmission from humans to mosquitoes and for evaluating the direct transmission-blocking effects of a vaccine.

## 1. Background and introduction

Human malaria is caused by infection with a mosquito-transmitted parasite. Various vector control methods and anti-malarial drugs are now available to decrease transmission and cure infections, but universal coverage with these interventions may be insufficient to interrupt transmission in areas where potential transmission intensity is very high [1,2], where the mosquitoes are refractory to vector control [3], or where operational constraints make it too difficult or expensive to achieve very high intervention coverage levels [4,5]. An effective malaria vaccine would complement existing technologies and could help to bring about an end to malaria [6]. Several malaria vaccines are now in development [7], but it is clear that evaluating the impact of such a vaccine is a complex endeavour [8]. One type of promising vaccine affects the sexual stages of the malaria parasites and reduces the probability that they will infect mosquitoes [9]. There is active debate about the appropriate way to evaluate these sexual-stage transmission-blocking vaccines. Though reductions in the incidence of clinical malaria are arguably the most important endpoint of any vaccine trial, intermediate entomological endpoints may be used to evaluate the direct population-level effects of a vaccine on transmission [10]. Such information is highly useful in making decisions about how and where to deploy a vaccine. Here, using mathematical models, we simulate parasite transmission from humans to mosquitoes, and we develop methods for accurately measuring transmission rates and the entomological outcomes of sexual-stage transmission-blocking vaccines in clinical trials.

Transmission of parasites from humans to mosquitoes involves uptake of at least one mature male and one mature female gametocyte in a bloodmeal, which form gametes and which following fertilization develop into a zygote, the ookinete. The motile ookinete penetrates the midgut where it transforms into a sessile oocyst [11]. To become infectious, an infected mosquito must survive long enough for the oocyst to rupture releasing sporozoites which must migrate through the mosquito haemocoel to reach the salivary glands; this process takes several days [11]. Sexual-stage transmission-blocking vaccines inhibit this development when antibodies present in human blood act against targets on the gametocyte, gamete, zygote or ookinete [12].

The efficacy of a sexual-stage transmission-blocking vaccine may be assessed by measuring changes in the rates that humans infect mosquitoes. Though this quantity describes a basic part of malaria transmission, methodologies for measuring it remain poorly developed for evaluation in field studies. Greater attention has been given to estimating the rate that humans are exposed to malaria, called the entomological inoculation rate (EIR), the number of infectious bites received per person per unit time [10,13]. Measuring transmission from human populations to mosquitoes in natural settings is an important complement to measuring EIR, but there are a number of factors that must be considered for proper evaluation of a transmission-blocking vaccine.

Transmission from a single human to mosquitoes can be measured by allowing mosquitoes to feed on humans or on human blood, and studies of this type are an indispensable part of understanding transmission [14]. In measuring infection rates of mosquitoes, it becomes possible to infer the probability a parasite is transmitted from a single infectious human to a mosquito and the proportion of mosquitoes in a population that become infected after biting a human. At a population-level, the corresponding quantity is the proportion of mosquitoes that would become infected after blood feeding on any human, and it is called the net infectiousness of humans, and often denoted by *κ* [15,16]. It is essentially impossible to measure this quantity directly in mosquito populations. Instead, it must be inferred by catching mosquitoes and examining them for oocysts or sporozoites [17]: the prevalences of infection with parasites at these developmental stages are called, respectively, the oocyst rate and the sporozoite rate. If the reservoir of malaria parasites in humans remained unchanged, a transmission-blocking vaccine would reduce the fraction of mosquitoes that successfully established oocyst infections in mosquitoes and, by extension, that reach the sporozoite stage.

An important problem is that the relationship between *κ* and either oocyst or sporozoite rates while simple in theory, is far more complicated in reality with both mosquito and parasite populations fluctuating over time. The prevalence of malaria parasites in mosquitoes is affected by highly heterogeneous feeding rates among individuals and among households, and the variable infectiousness of individual humans as gametocyte densities fluctuate and natural levels of human immunity wax and wane [18,19]. It is also affected by heterogeneity in the susceptibility or refractoriness of mosquitoes to infection [20–22].

Simple formulae have been developed to understand these relationships in constant populations [23], but these formulae rely heavily on the assumption that mosquito populations are constant over time with a stable age distribution. However, spatio-temporal fluctuations in mosquito densities commonly alter the age-structure of mosquito populations [24]. Owing to the time the parasite needs to develop, the older a mosquito, the more likely it is to be infected or infectious. Therefore, emergence of a large cohort of young adult mosquitoes, driven by seasonal rainfall for example, would instantly reduce the sporozoite rate in a mosquito population. If recruitment of new mosquitoes slowed down or stopped for a time, such as during a dry season (e.g. malaria transmission in the Sahel), the sporozoite rate would tend to increase as mosquitoes both aged and became infected. It follows that the prevalence of parasite infection in mosquitoes is as strongly affected by the age distribution of mosquitoes as it is by *κ*. To measure the effects of a sexual-stage transmission-blocking vaccine, it would be necessary to have accurate measures of *κ* before and after vaccination. To evaluate efficacy of a sexual-stage transmission-blocking vaccine, it is tempting to forgo measurement of *κ* and simply compare mosquito infection rates before and after mass vaccination. However, the precision and accuracy of such methods have not been rigorously evaluated under the conditions that prevail in nature.

In many studies, variability in mosquito population densities is either ignored or treated as a nuisance, or noise. In fact, noise is an interesting phenomenon that can be an important driver of long-term variation in populations [25,26], but there are many types of noise distinguished by their ‘colour’ [27–30]. While it is, perhaps, more common to think about noise as it impacts stock market performance, and the expected growth in the value of one's retirement portfolio, coloured noise is also a challenge for ecology and epidemiology. White noise is a term used to describe a time series that is approximately equally variable in any sampling frame. Blue noise describes time series with more variation at small sampling frames—the biggest differences are close together. Red noise describes time series in which the variation grows with the size of the sampling frame, such that the larger the temporal window, the more variation. When a noisy process is driving a system with strong intrinsic dynamics, the results can affect many different properties of such systems. Numerous previous studies in ecology have looked at the effects of ‘memory’ within dynamic systems; a concept mostly described using ‘coloured’ noise [31,32]. Several of these studies show how emergence rates with ‘blue noise’ [29]—i.e. noise that is negatively auto-correlated at short timescales—can interact with the intrinsic dynamics to give rise to different colours of noise [25,33], specifically the more commonly observed ‘red noise’—i.e. noise that is positively auto-correlated at long timescales [25,33–36]. Population dynamics that are strongly seasonal often exhibit ‘red noise’, while those that are strongly influenced by density dependence and are at carrying capacity may display ‘blue noise’. Here, following their lead, we consider the effects of coloured noise on the ability to measure transmission in mosquito populations, and we also consider the problem posed by measuring the effects of sexual-stage transmission-blocking vaccines in natural populations.

## 2. Material and methods

### 2.1. Simulated mosquito population sampling

**Figure 1.**
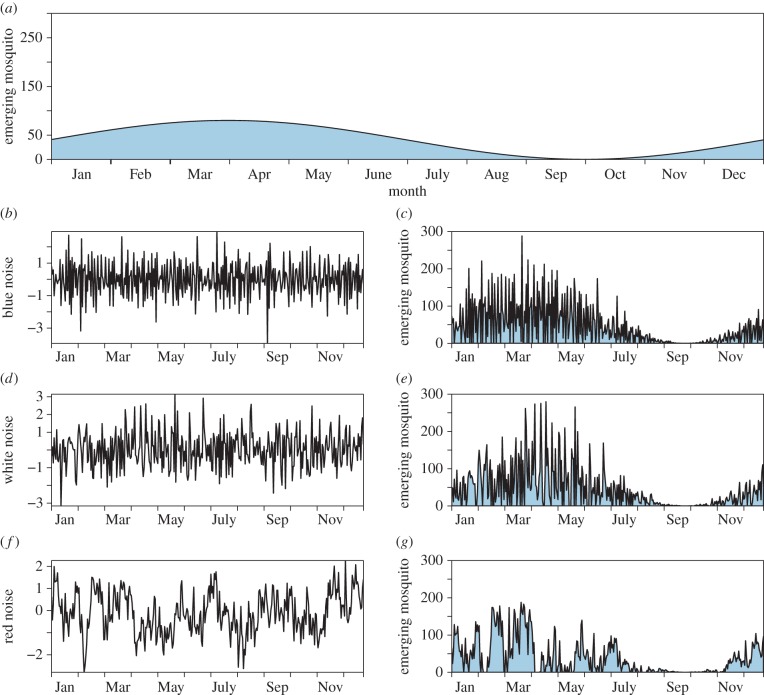
Mosquito emergence with coloured noise. A baseline seasonal mosquito emergence (*a*) is modulated by coloured noise. The left panels (*b,d,f*) display a realization of blue noise (Hurst parameter = 0.01), white noise (Hurst parameter = 0.5) and red noise (Hurst parameter = 0.99), respectively. The resulting effect incorporating these noises with mosquito emergence is displayed in the corresponding right panels (*c,e,g*).

**Figure 2.**
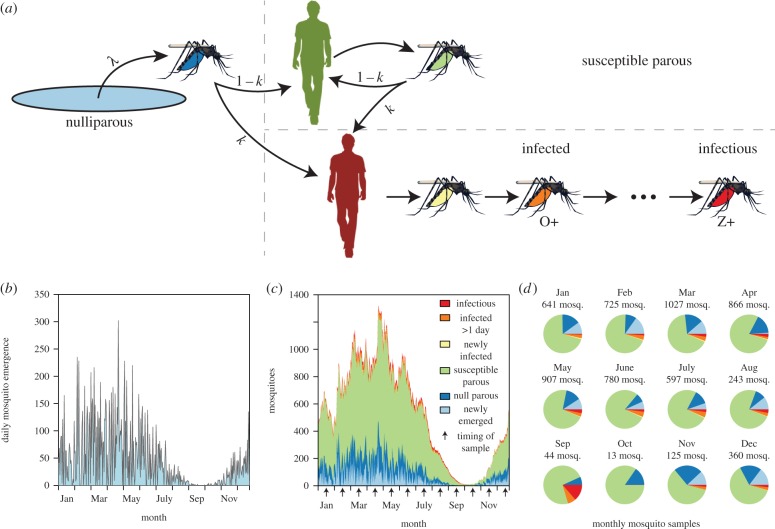
Mosquito life/infection history. (*a*) Mosquitoes emerge at rate *λ* (light blue) and are nulliparous (dark blue) until they take their first bloodmeal. They are then classified as susceptible parous or exposed parous depending on the infection status of the individual they feed on and the success of the parasite (infection rate from bloodmeals is *κ*). Non-exposed parous mosquitoes (light green) continue taking bloodmeals until they either die or become exposed to the malaria parasite. Exposed mosquitoes (yellow) progress sequentially through the states of infection from infected (orange, assumed to be observable on day 2 of exposure) to infectious (red, on day *n*). (*b*) A realization of mosquito emergence, (*c*) a realization of the entire mosquito population's parity and infection status over the course of a single year (simulation was run for 2 days and the second year was taken to initialize each state). (*d*) Variation in the composition of simulated mosquito captures depending on which month the sample was taken from.

A set of algorithms was developed to simulate field studies to estimate *κ* and changes in *κ* in variable mosquito populations involving three elements: (i) recruitment of adult mosquitoes (aka emergence from aquatic habitats) was simulated with a simple seasonal signal modified by coloured noise (figure 1); (ii) after emergence, adult blood feeding, infection and survival were simulated using Ross–Macdonald assumptions (figure 2); and (iii) a study involving mosquito population sampling was simulated with properties that would mimic observed sampling variance (figure 1). These are described in greater detail in the following paragraphs.

Recruitment of adult female mosquitoes was simulated by comparing constant and canonical sinusoidal seasonal recruitment patterns to the same patterns combined with a fractional Brownian noise [37]: the number of adult female mosquitoes that were recruited into a population, per human, on a given day was denoted *λ_t_*. Formally, fractional Brownian noise can be defined as the derivative of fractional Brownian motion, a non-Markovian Gaussian process. If *B_H_*(*t*) denotes a fractional Brownian process, *B_H_*(*t*) has mean zero and covariance function2.1
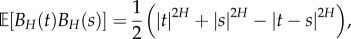
where *H*, the Hurst parameter, is a real number between 0 and 1. Values of *H* between 0 and 0.5 produce ‘blue’ noise, while values between 0.5 and 1 produce ‘red’ noise. If *H* = 0.5, the process reverts to standard Brownian motion (‘white’ noise). The population density of mosquitoes tended to follow the canonical seasonal pattern, but because of the added noise, any particular realization tended to differ from the canonical seasonal signal (figure 1).

After emerging, adult population and infection dynamics were simulated day by day assuming that a proportion of mosquitoes survived each day (*p*), that a fraction of mosquitoes took a blood meal each day (*f*), that a fraction of those bloodmeals were from humans (*Q*) and that a fraction of human blood feeding mosquitoes became infected with parasites (*κ*). We assume here that infected mosquitoes are oocyst positive after 1 day. This assumption could be relaxed (e.g. shifted to day 2 of infection), which would correspondingly result in less precise estimates due to fewer mosquitoes surviving the extra day. After *n* days, infected mosquitoes became infectious (i.e. *n* is the extrinsic incubation period (EIP)). A set of equations tracked the population density of mosquitoes in each relevant state: including those that were nulliparous (*N_t_*), having never taken any bloodmeal; those that were parous but uninfected; those that were infected with parasites *i* (up to *n*) days ago 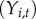 and those that were sporozoite positive 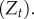 The total number of adult mosquitoes on day *t* was denoted *M_t_*.

To have a measure of the accuracy and precision of estimates of *κ*, we simulated a study of transmission under three different vector sampling regimes: monthly (which is a common sampling interval in the field), bi-weekly (which would be considered intense sampling in practice) and alternating days (which would be almost impossible to achieve in the field due to limited resources). It is also important to note that we assume that our sampling effort does not affect the population size (i.e. sampled mosquitoes are replaced into the population to potentially be sampled again later). For each regime, we simulated sampling by two different teams initiating their sampling at offset times throughout the year. Teams were assumed to be equally capable of sampling. For the monthly sampling regime, for example, one team samples on the 15th of each month and the other samples on the last day of each month. The distribution of mosquitoes among houses tends to follow a negative binomial distribution, so mosquito catch data from a single household on a single night was simulated by drawing a random variate from a negative binomial distribution [38]: the expected number of mosquitoes was given by 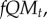 but the variance of this distribution was given by the parameter *α* reflecting among household variability in the number of mosquitoes present.

Other than *λ_t_*, all of the parameters that govern the simulation are set to constant values. For our initial simulations, we set 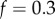 (the fraction of mosquitoes that take a blood meal in a day), 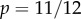 (the probability a mosquito survives a day), 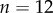 (the EIP), *Q* = 1 (the per cent of mosquitoes that feed on humans) and 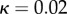 (the per cent of human blood meals that result in infected mosquitoes). Under the set of equations governing our system, *Q* is inseparable from *κ*, and can thus not be individually estimated within our framework. For simplicity, we assume 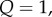 but could as easily remove that assumption and reformulate our question as estimating 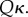 It is also important to note that in specific settings, *Q* could be estimated through, for example, identifying the prevalence of human DNA within bloodmeals [39,40].

### 2.2. Precision and accuracy of the estimates of *κ*

We used the simulated values of three commonly used statistics as a basis for estimating *κ* and changes in it: parity in the mosquito population, the fraction that had ever laid an egg batch, which by our model assumptions includes any mosquito that has taken at least one blood meal; the oocyst rate, that is in our model the fraction of mosquitoes that had ever been infected and that had survived at least one full day—oocyst positive mosquitoes in our model would include all mosquitoes that were sporozoite positive regardless of whether any oocysts remained; and the sporozoite rate (figure 2). We evaluate statistical bias in our estimators (the difference between the expected value of the estimator and the true population parameter) using the mean *κ* of the simulations. At equilibrium, assuming a constant mosquito emergence rate, Macdonald's model suggests that the sporozoite rate, oocyst rate and parity would reach equilibrium values that are functions of the parameters *f*, *n*, *p* and *κ* (electronic supplementary material, table S1). Estimating *κ* from either empirical measures of sporozoite rates or oocyst rates alone requires assuming the values of four of the five bionomic parameters: here, we assume the known parameters were *f*, *Q*, *n* and *p*. Empirical measures of any two of sporozoite rate, oocyst rate or parity would require assuming three of the five bionomic parameters: here, we assume the known parameters were *Q*, *n* and *p*. Finally, estimating *κ* given empirical measures of all three rates only requires assuming the values of only two mosquito bionomic parameters: here, we assume a known value of *Q* and *n* (table 1).

**Table 1.** Formulae for various estimates of *κ* depending on the data collected and parameters assumed known. These equations hold under the assumption *Q* = 1. Alternatively, without this assumption, these equations represent six different estimates of *Qκ*.

estimate	parameters assumed	data collected	other parameters estimated	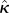
1	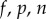	sporozoite rate: 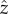	none	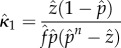
2	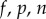	oocyst rate: 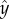	none	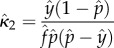
3	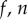	sporozoite rate: 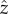 ; oocyst rate: 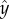	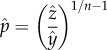	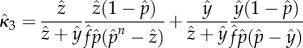
4	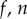	sporozoite rate: 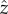 ; parity: 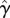	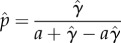	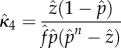
5	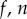	oocyst rate: 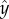 ; parity: 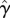	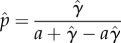	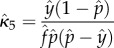
6	*n*	sporozoite rate: 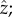 oocyst rate: 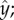 parity: 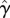	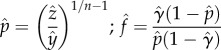	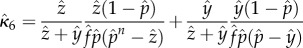

To measure a change in *κ*, we simulated changes following a mass vaccination campaign with a sexual-stage transmission-blocking vaccine by comparing the estimated value of *κ* after a 25%, 50% or 80% drop from its baseline value. By comparison, we also measured changes in *κ* by taking the sporozoite rate before and after the vaccination and the oocyst rate before and after vaccination.

All simulations and calculations were performed using R v. 3.1.1 [41]. Fractional Brownian noise was calculated using fArma [42]. A maintained version of all code used is available at http://github.com/bcreiner/measuring_kappa.

## 3. Results

### 3.1. Sporozoite and oocyst rates

Sporozoite and oocyst rates estimated over a simulated year by two different *in silico* sampling teams with offset timing schedules differed. We compared both the correlation between oocyst and sporozoite rates and the relative accuracy of these rates across the different sampling regimes. Using the simulation parameter values and the equations from the electronic supplementary material, table S1, at equilibrium, the average estimated oocyst rate equalled 5.7% and the sporozoite rate equalled 2.2%. When the mosquito emergence was governed by red noise, differences in the paired teams' estimates decreased as sampling effort increased (electronic supplementary material, figure S1). When the samples were taken monthly, even though the two teams sampled only 15 days apart, the estimated rates were not strongly correlated (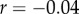 and 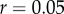 for oocyst and sporozoite rates, respectively), but the estimates appeared unbiased (mean oocyst rate = 5.62% (s.d. = 0.85%), mean sporozoite rate = 2.11% (s.d. = 0.52%), electronic supplementary material, figure S1). Results were similar (though more precise) when sampling increased to every other week. Conversely, when samples were taken every other day (which again is an extremely intense, relatively infeasible sampling regime) the estimates appeared strongly correlated (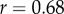 and 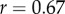 for oocyst and sporozoite rates, respectively). For white and blue noise (electronic supplementary material, figures S2 and S3, respectively), the estimates also increased in correlation as sampling intervals decreased, are likewise unbiased, and have relatively diminishing imprecision compared with their red noise counterpart.

### 3.2. Estimating *κ*

Here and throughout the remainder of the article, we simulated and analysed sampling done by a single team (i.e. either sampling on the 15th of every month for 1 year (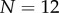), the 1st and 15th of every month (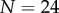) or on odd-numbered days (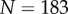). To assess the impact of sampling effort, we plotted both the resulting estimates calculated from a single collection per sampling effort and the estimates calculated when each sampling effort assumed five collections at five uncorrelated homes (i.e. 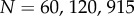).

To estimate *κ*, it was necessary to have some knowledge of at least one other parameter affecting oocyst or sporozoite rates. When parity, oocyst and sporozoites rates were all used, only one other parameter was required. When only two of these statistics were used, independent knowledge of two other parameters was required, and when only one of these was used, independent knowledge of three parameters was required. Assuming perfect knowledge of the EIP, or the interval from infection to infectiousness within the mosquito (*n*, here set to 12), and using estimates of parity and the oocyst and sporozoite rates derived from the sampled data, as well as the sixth method of estimation given in table 1 (i.e. the method that incorporates all available information), we plotted the resulting estimates of the transmission intensity, *κ*.

**Figure 3.**
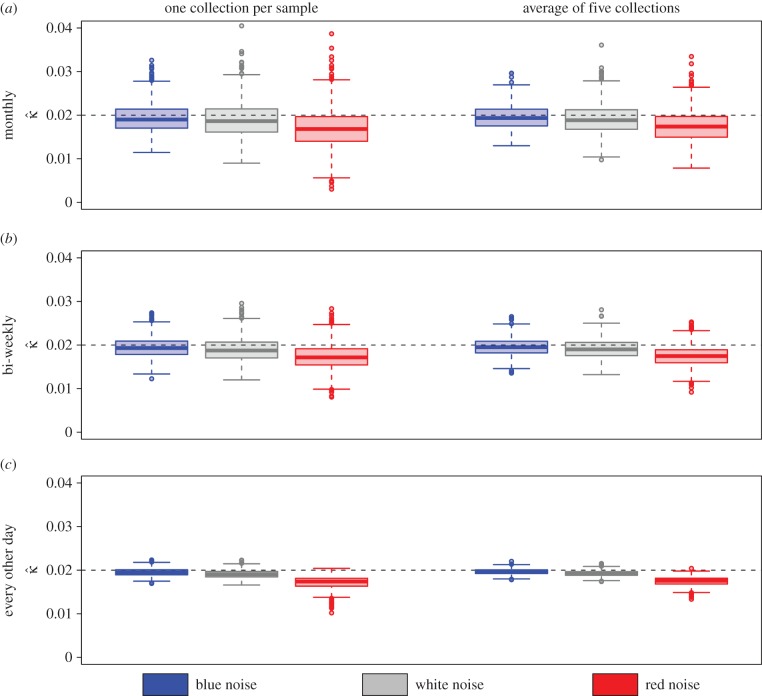
Estimating *κ* with 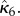 Estimates of *κ* using the sixth estimation method are plotted for simulations where emergence incorporated either blue, white and red noise and collections were done every month (*a*), bi-weekly (*b*) or every other day (*c*). Sampling error is assessed by contrasting estimates based on a single collection (left box plots) versus the average of five collections all done on the same night (right box plots). True value indicated by black dashed line.

Consistent with the estimates of the oocyst and sporozoite rates, estimates of *κ* had larger variation when sampling occurred every month when compared with bi-weekly or alternating day sampling regimes (figure 3). Also similar to the estimates of the oocyst and sporozoite rates, the variation in the estimates was largest for red noise and smallest for blue noise. There appeared to be a small bias in the estimated values for simulations where emergence was driven by either white or red noise (estimates are lower than expected), with the bias being larger for simulations with red noise.

Estimation of *κ* when less information was assumed to be collected (and thus more parameters' values must be assumed to be known) resulted in some similar patterns across Hurst parameters and sampling regimes in that variation in the estimates was largest for red noise and when there were monthly samples (electronic supplementary material, figures S4–S8). It must be noted here that these alternative estimation approaches were calculated using exact values of parameters that in practice would need to be estimated, so over interpretation of the relative accuracy and precision between estimates plotted in figure 3 and electronic supplementary material, figures S4–S8 is inapt. However, it is interesting to note that estimates based on sporozoite rate, which naturally will be harder to accurately estimate given its small value, but which assume knowledge of at least one additional parameter, are less accurate (electronic supplementary material, figures S4 and S5). This decrease in accuracy is most notable when data are collected on both parity and sporozoite rate (electronic supplementary material, figure S7) where the fact that parity rate estimates are raised to the power of *n* + 1 causes estimates of *κ* to be incredibly inaccurate (and in the case of red noise, relatively useless).

### 3.3. Changes in *κ*

**Figure 4.**
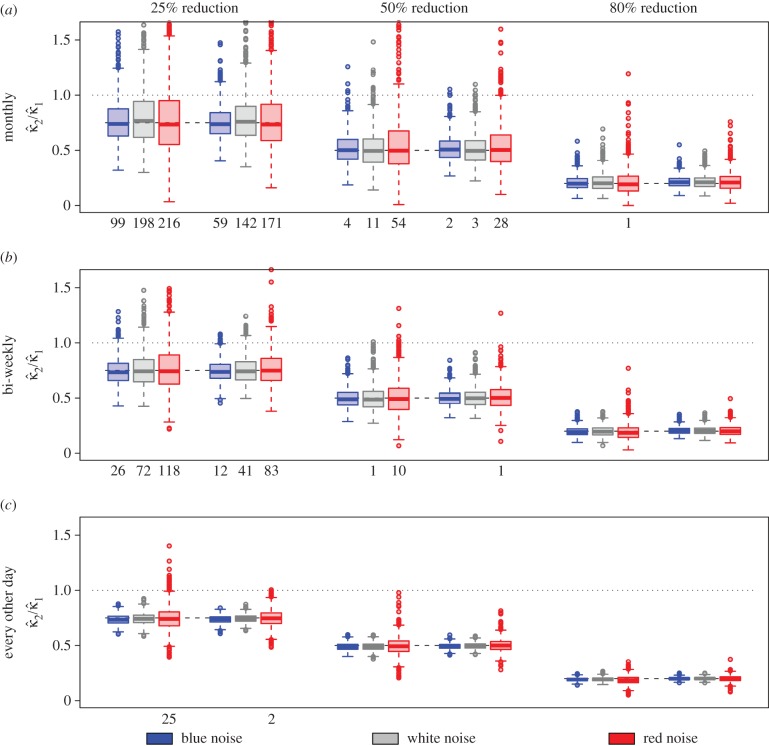
Estimating reduction in *κ* with 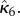 Reduction in transmission intensity using the sixth estimation method was simulated for three scenarios, 25% reduction (left plots), 50% reduction (middle plots) and 80% reduction (right plots). For each scenario, estimates are plotted for simulations where emergence incorporated blue, white, or red noise and collections were done every month (*a*), bi-weekly (*b*) or every other day (*c*). Sampling error is assessed by contrasting estimates based on a single collection (left box plots) versus the average of five collections all done on the same night (right box plots). True value indicated by blue dashed line, while ‘no difference’ (i.e. 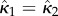) indicated with black dotted line. Out of 1000 simulations, the number of simulations where no decrease in transmission intensity was detected is listed below the corresponding box plot.

Our relative ability to estimate a reduction in *κ* from one year to the next depended on both the size of the reduction in *κ* as well as the colour of the noise driving the emergence rate (figure 4). In all cases, the estimated change in *κ* appeared to be unbiased (unlike actual estimates of *κ* for some noise regimes). However, in certain simulation scenarios, the variation in estimates of the change in *κ* was so large that it obscured the presence of an effect. In one extreme, when *κ* was reduced by 25%, monthly samples were taken and red noise drove mosquito emergence, 21.6% of the simulations failed to result in estimates that indicated any reduction in transmission intensity at all (figure 4*a*). At the other extreme, when there was an 80% reduction in transmission intensity, samples were taken every other day and blue noise drove mosquito emergence, not only were the estimates unbiased (mean ratio is 0.1987 versus true ratio of 0.2), but also the variance in the estimate was extremely small (s.d. = 0.012). There was a small reduction in variance when estimates were calculated assuming samples were based on the average of five collections, but the reduction was as large as when samples were more spread. In particular, monthly samples based on five collections once each month on the 15th (i.e. 120 collections on 24 distinct days over 2 years; figure 4*a*) had a higher variance than bi-weekly samples based on single collections on the 1st and 15th of each month (i.e. 48 collections on 48 distinct days over 2 years; figure 4*b*).

As with estimates of *κ*, estimates of the change in *κ* using less collected information but assuming the exact value of more of the parameters governing the simulations creates similar patterns (electronic supplementary material, figure S9–S13). Estimates that did not use oocyst rates were extremely inaccurate (electronic supplementary material, figures S9 and S12), while other estimates appeared similar in power to those based on 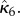 It is interesting to note that in spite of the inaccuracy in sporozoite rates, estimates of the change in *κ* using oocyst and sporozoite rates (electronic supplementary material, figure S11) performed better than estimates using oocyst rates and parity (electronic supplementary material, figure S13). Estimates of *κ* itself followed the opposite pattern (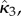 electronic supplementary material, figure S6; and 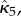 electronic supplementary material, figure S8, respectively).

Estimates of the change in *κ* made by calculating either the change in the oocyst rate (electronic supplementary material, figure S14) or the sporozoite rate (electronic supplementary material, figure S15) were biased, consistent with the above observation that when more information is used, the estimates perform better. In all cases, the size of the reduction was underestimated (as indicated by the median ratio of estimated *κ*s always being higher than the corresponding dashed lines in electronic supplementary material, figures S14 and S15). In addition to being biased, the variation in the ratio of sporozoite rates was larger than the variation in estimates of *κ* in every scenario. Alternatively, the variation in the ratio of oocyst rates was smaller in every scenario than the corresponding ratios of estimates of *κ*, in part, because there was less time for the noise in recruitment to change the age distribution of the mosquito population. It is, however, important to note that our direct estimates of *κ* assumed knowledge of *n*, while the ratio of oocyst or sporozoite rates required no assumptions of any of the parameters of the model.

## 4. Discussion

Measuring transmission from mosquitoes to humans is a challenge, but this study and the methods we have developed show it is possible to estimate net infectiousness of human populations with accuracy and precision even when mosquito populations fluctuate in some unknown way. Proper evaluation of different methods requires modelling fluctuating mosquito populations, but this has been problematic because of the difficulty of simulating variability in any systematic way. We have solved that problem using fractional Brownian processes to generate noise with different colours to represent fluctuations in mosquito populations. The analysis also suggests that the colour of the noise in mosquito population dynamics will affect both the precision and accuracy of the estimates of *κ*, but that these problems can be overcome to some extent by sampling the population sufficiently frequently throughout the season and using multiple measures of infection. The most unbiased method for measuring changes in *κ* accurately requires simultaneously measuring parity, oocyst rates and sporozoite rates. We have also shown that using crude oocyst rates or sporozoite rates to estimate changes in *κ* gives biased results. Using estimators that capture different information about the age of populations—parity, oocyst rates and sporozoite rates, which convey some information about the age of the population—removes much of the bias introduced by environmental and sampling noise.

There are also several caveats that could limit the applicability of this study to actual field conditions. First, some *a priori* knowledge of the EIP (or perhaps of some other parameter) is necessary, regardless, from other studies of the mosquito populations. Second, these methods assume that most of the mosquito bionomic parameters are constant, while focusing only on variability in the rate of recruitment. In real populations, mosquito survival and blood feeding rates, parasite development rates, and *κ* almost certainly vary seasonally. Adding variability in these other parameters could further reduce the precision and accuracy of estimates, which is of sufficient concern that we have not yet made any specific recommendations about the proper way to power a study. Finally, inferring the colour of a the noise of a dynamic process is a difficult task and is still an active filed of research. Simple investigation of the power spectrum of a process may yield some insight, and in recent years more complex estimation approaches can incorporate more complex dynamics [43,44].

Previous modelling studies have looked at the role of coloured noise in generating complicated population dynamic time series [25,26]. Here, we have explored the ways that coloured noise affects our ability to measure epidemiologically relevant processes occurring in malaria vector mosquito populations. This result is consistent with previous simulation modelling studies that illustrate the difficulties of estimating mosquito survival in variable populations [45]. Our study also highlights the primitive state of metrology for entomological aspects of malaria ecology and epidemiology. Formal analysis of mosquito population variability is rarely done, and few studies have simultaneously considered sampling variance, local spatio-temporal heterogeneity, and seasonal or environmental variance and its effects over time on the ability to measure various quantities of interest [45,46]. Similarly, though there is substantial evidence that mosquito catch data follow a negative binomial distribution [38,47,48], and though there is theory supporting the use of negative binomial distributions for analysing mosquito catch data [49], such methods are rarely used in practice. There are also remarkably few examples of field data in which parity and both sporozoite and oocyst infection rates have been collected. Two landmark malaria controls studies in Africa, Garki [50] and Pare-Taveta collected only sporozoite rate and parity and a review by Killeen *et al.* [16] identified only six studies that had collected these three components. Our simulation studies suggest that it is possible to measure many quantities associated with mosquito populations with precision and accuracy, but that much greater care must be taken to develop methods for estimation, and appropriate study designs and sample sizes. We have shown that it is possible to measure transmission accurately and precisely in randomized control trials with the proper study design, the proper estimation tools and with sufficient sampling intensity. Variability in mosquito populations is, however, real and it can have a large effect on the outcome and conclusions of any trial, especially if the assumed effect sizes within the trial are moderate.

Mosquito population density and mosquito behaviours are an uncontrolled variable that affect the outcomes of any malaria study. In statistical terms, all studies of malaria are quasi-experiments and the results of any trial must be interpreted with proper entomological measures as a covariate. Far from arguing against inclusion of entomological measures, this study suggests that, although it is difficult to measure transmission entomologically, it can be done properly given enough forethought and effort. At the present time, parity and sporozoite rates are standard measures, but given modern high throughput molecular methods, oocyst rates could easily be added to the mix using the same sampled mosquitoes providing a natural complement to overcome some of the problems associated with fluctuating mosquito populations. The sensitivity of this approach would be further improved by age grading techniques for anophelines which could be integrated as molecular [51] or stand-alone approaches [52]. Here, age can be inferred from both parity but also the development of the malaria parasite. Many of the techniques applied here to malaria would be generally applicable to any vector-borne pathogen where age can be inferred from some aspects of pathogen development. This study suggests that it is time to devote more resources and capacity to developing appropriate methods and appropriate sampling effort to getting the entomological measures right, which requires a combination of expertise in entomology, parasitology, statistics, mathematical models and experimental design.

## Supplementary Material

Supporting Online Material for Estimating malaria transmission from humans to mosquitoes in a noisy landscape
