# Supplementary material for: Estimating malaria transmission from humans to mosquitoes in a noisy landscape
Source: J R Soc Interface. 2015 Oct 6;12(111):20150478. doi: 10.1098/rsif.2015.0478 (PMC4614487; doi:10.1098/rsif.2015.0478)
Supplement: Supporting Online Material for Estimating malaria transmission from humans to mosquitoes in a noisy landscape [file rsif20150478supp1.pdf]

Supporting Online Material for

Estimating malaria transmission from humans to mosquitoes in a noisy landscape

Robert C. Reiner, Jr., Carlos Guerra, Martin J. Donnelly, Teun Bousema, Chris Drakeley, David L Smith

correspondence to: rcreiner@ucdavis.edu

	This quantity	Smith & McKenzie[16]	This paper
Mosquito density	M	$\frac{\lambda}{g}$	$\frac{\lambda}{1-p}$
Parity	P	$\frac{f}{g+f}$	$\frac{pf}{1-p(1-f)}$
Infected	Y	$\frac{fQ\kappa}{g+fQ\kappa}$	$\frac{pfQ\kappa}{1-p(1-fQ\kappa)}$
Oocycst rate (infected $\geq$ 1 day)	pY	$\frac{fQ\kappa}{g+fQ\kappa}\exp^{-g}$	$\frac{p^2 fQ\kappa}{1-p(1-fQ\kappa)}$
Sporozoite rate	Z	$\frac{fQ\kappa}{g+fQ\kappa}\exp^{-gn}$	$\frac{p^{n+1} fQ\kappa}{1-p(1-fQ\kappa)}$

Table S1: Formulas for various quantities if the recruitment rate of adult mosquitoes were constant. These formulas motivate the quantities of interest.

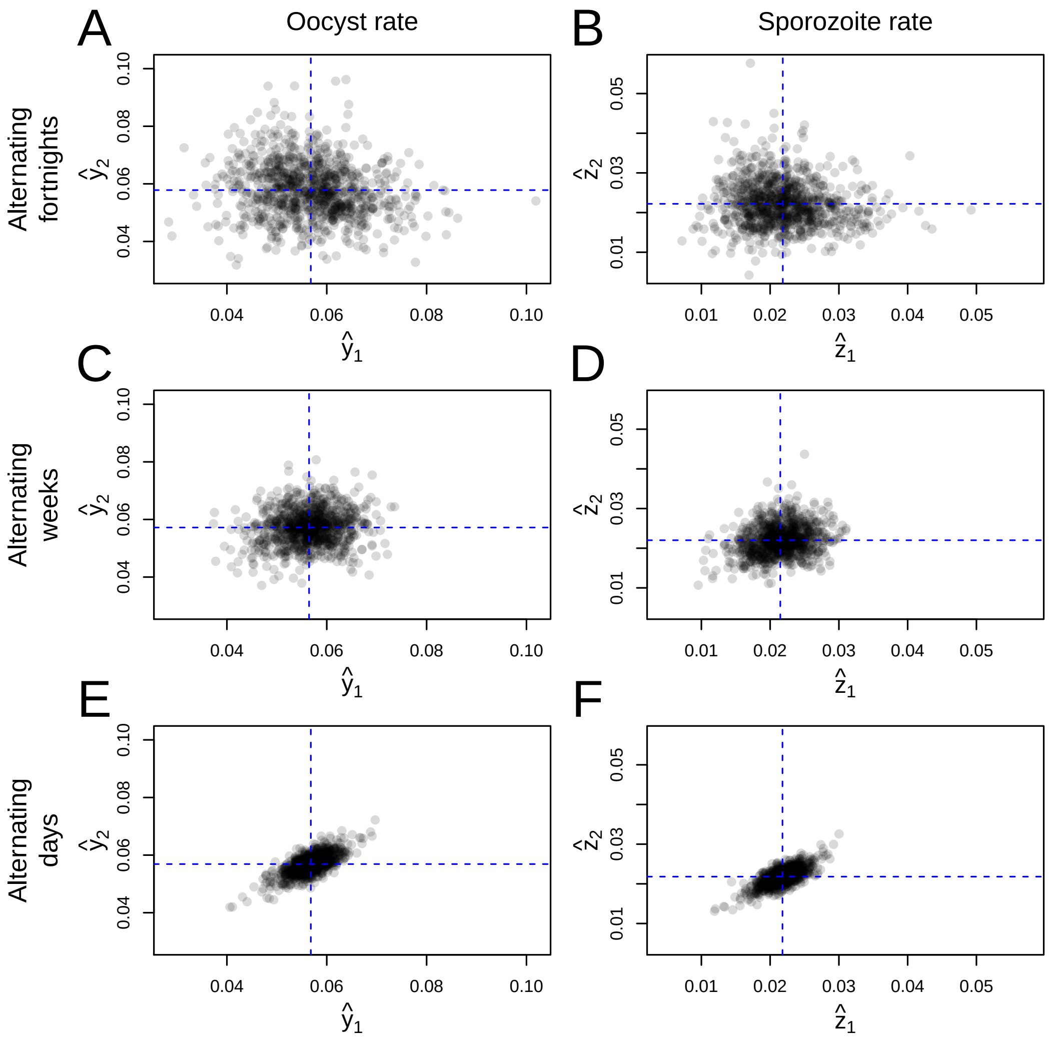

Fig. S1: **Estimated oocysts and sporozoite rate, red noise.** Estimates of the oocyst rate and sporozoite rate for monthly, bi-weekly or bi-daily collections contrasted against collections done on 15, 7 or 1 day earlier, respectively. Mosquito emergence here incorporated red noise. Equilibrium values indicated with blue dashed lines.

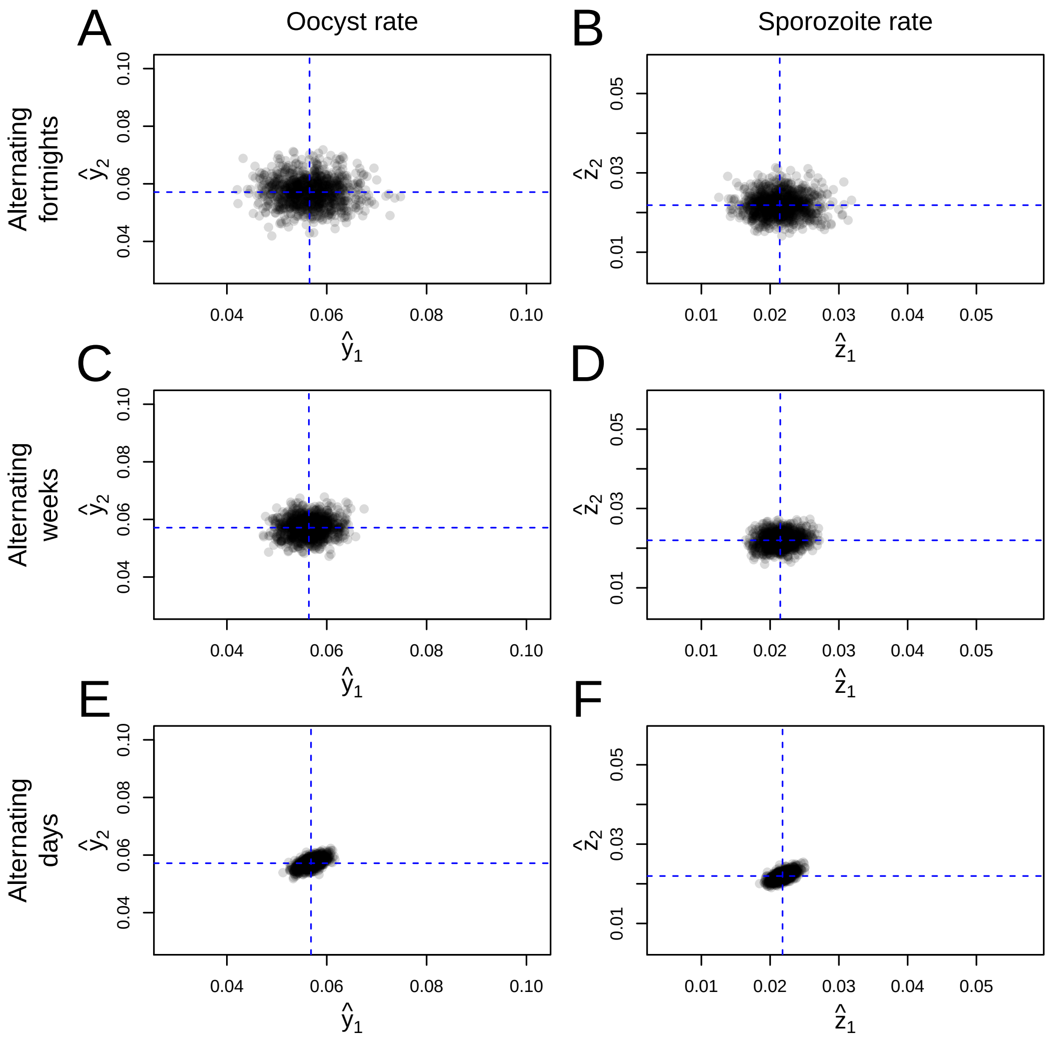

Fig. S2: **Estimated oocysts and sporozoite rate, white noise.** Estimates of the oocyst rate and sporozoite rate for monthly, bi-weekly or bi-daily collections contrasted against collections done on 15, 7 or 1 day earlier, respectively. Mosquito emergence here incorporated white noise. Equilibrium values indicated with blue dashed lines.

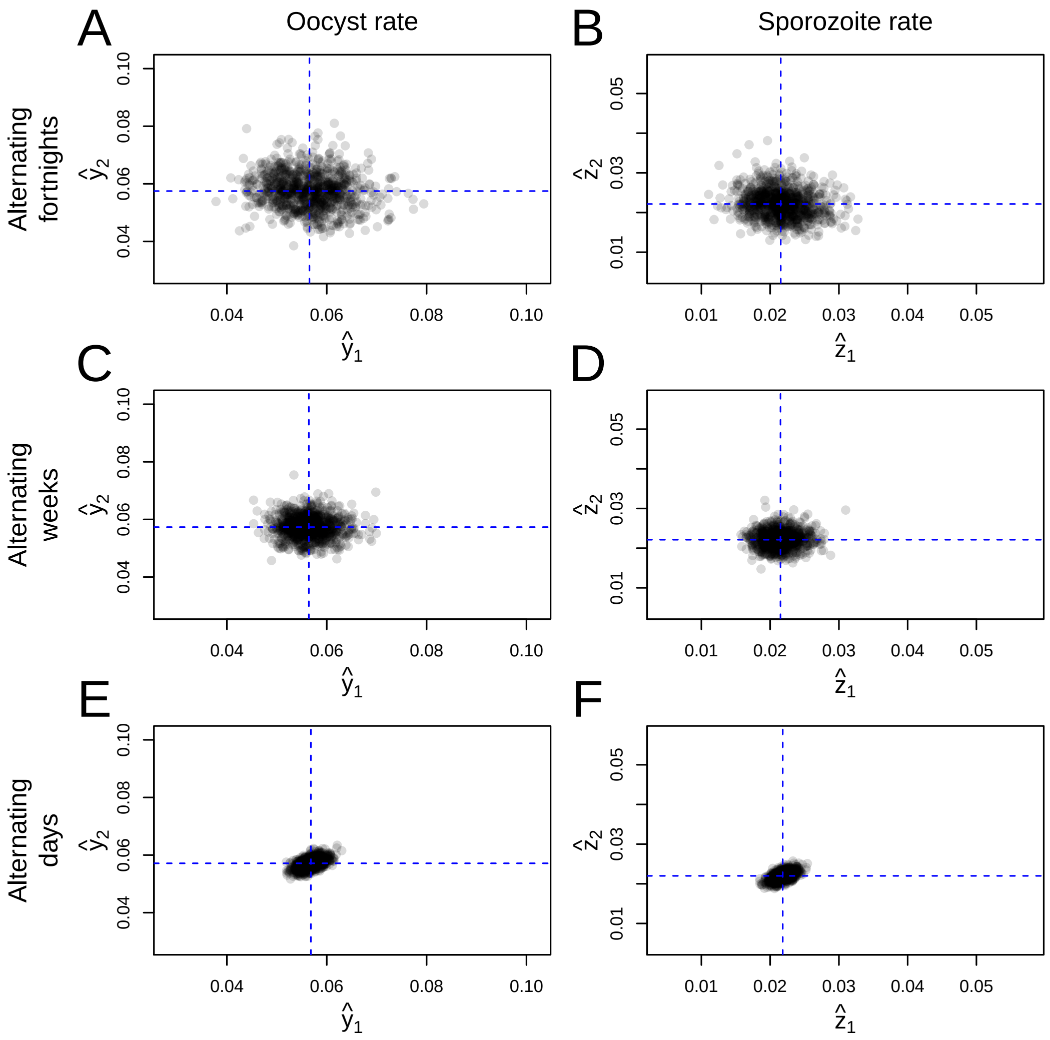

Fig. S3: **Estimated oocysts and sporozoite rate, blue noise.** Estimates of the oocyst rate and sporozoite rate for monthly, bi-weekly or bi-daily collections contrasted against collections done on 15, 7 or 1 day earlier, respectively. Mosquito emergence here incorporated blue noise. Equilibrium values indicated with blue dashed lines.

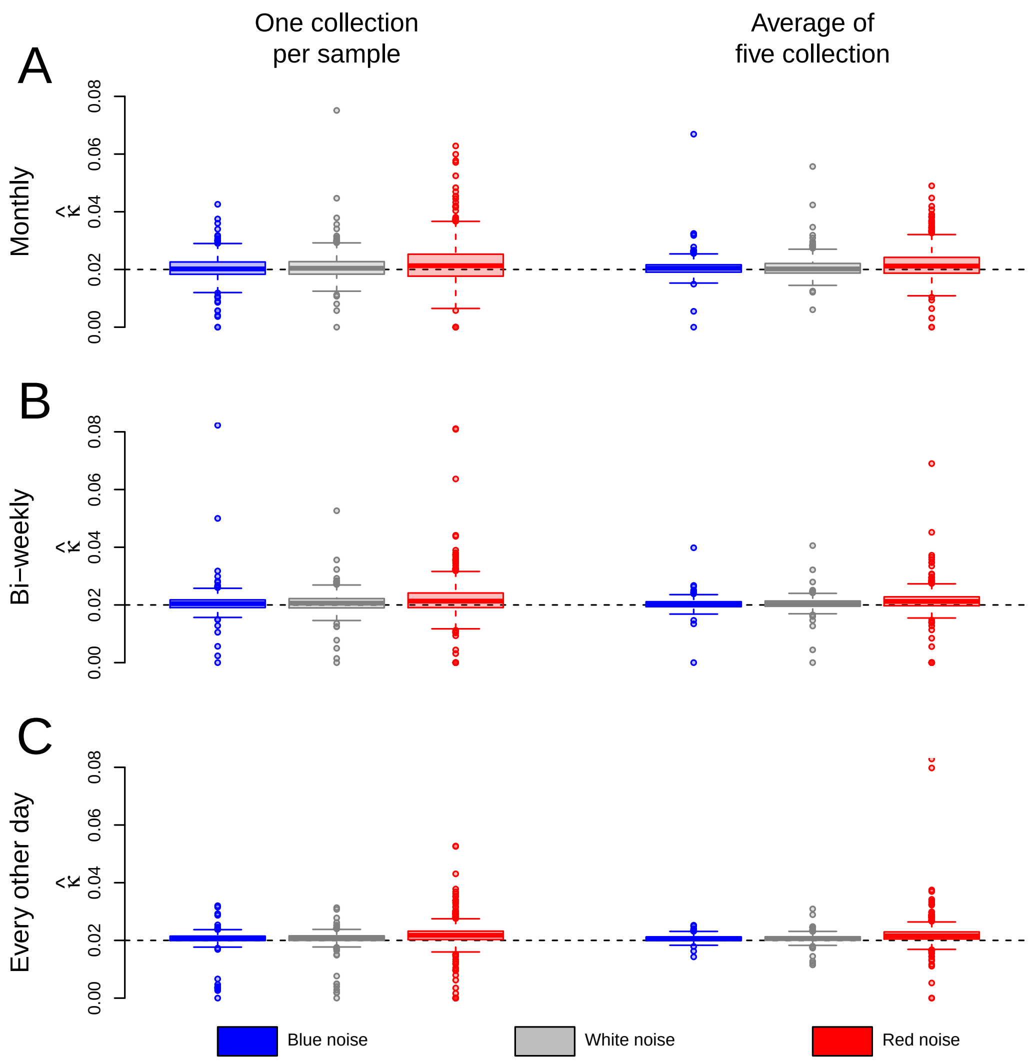

Fig. S4: **Estimating κ with $\hat{\kappa}_1$.** Estimates of κ using the 1st estimation method are plotted for simulations where emergence incorporated either blue, white and red noise and collections were done every month (panel A), bi-weekly (panel B) or every other day (panel C). Sampling error is assessed by contrasting estimates based on a single collection (left box plots) versus the average of five collections all done on the same night (right box plots). True value indicated by black dashed line.

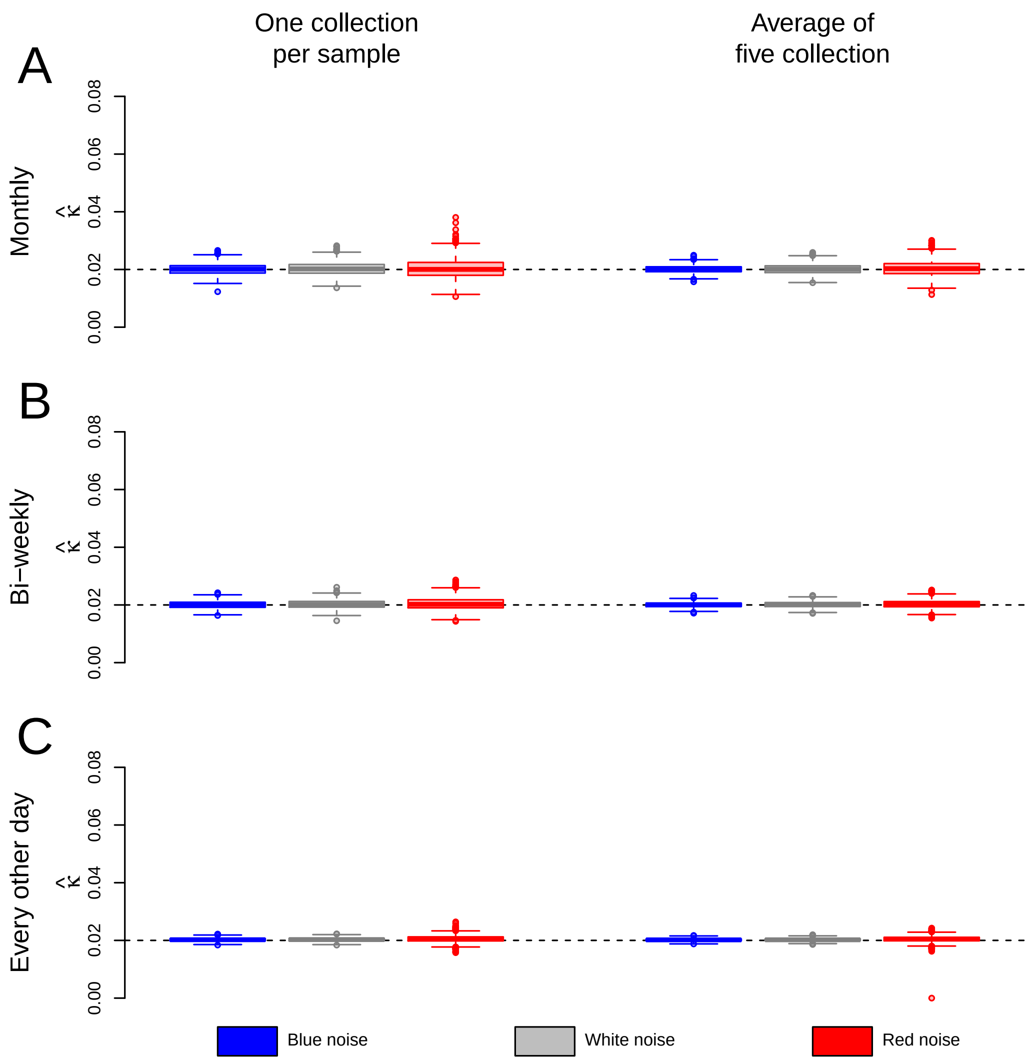

Fig. S5: **Estimating κ with $\hat{\kappa}_2$.** Estimates of κ using the 2nd estimation method are plotted for simulations where emergence incorporated either blue, white and red noise and collections were done every month (panel A), bi-weekly (panel B) or every other day (panel C). Sampling error is assessed by contrasting estimates based on a single collection (left box plots) versus the average of five collections all done on the same night (right box plots). True value indicated by black dashed line.

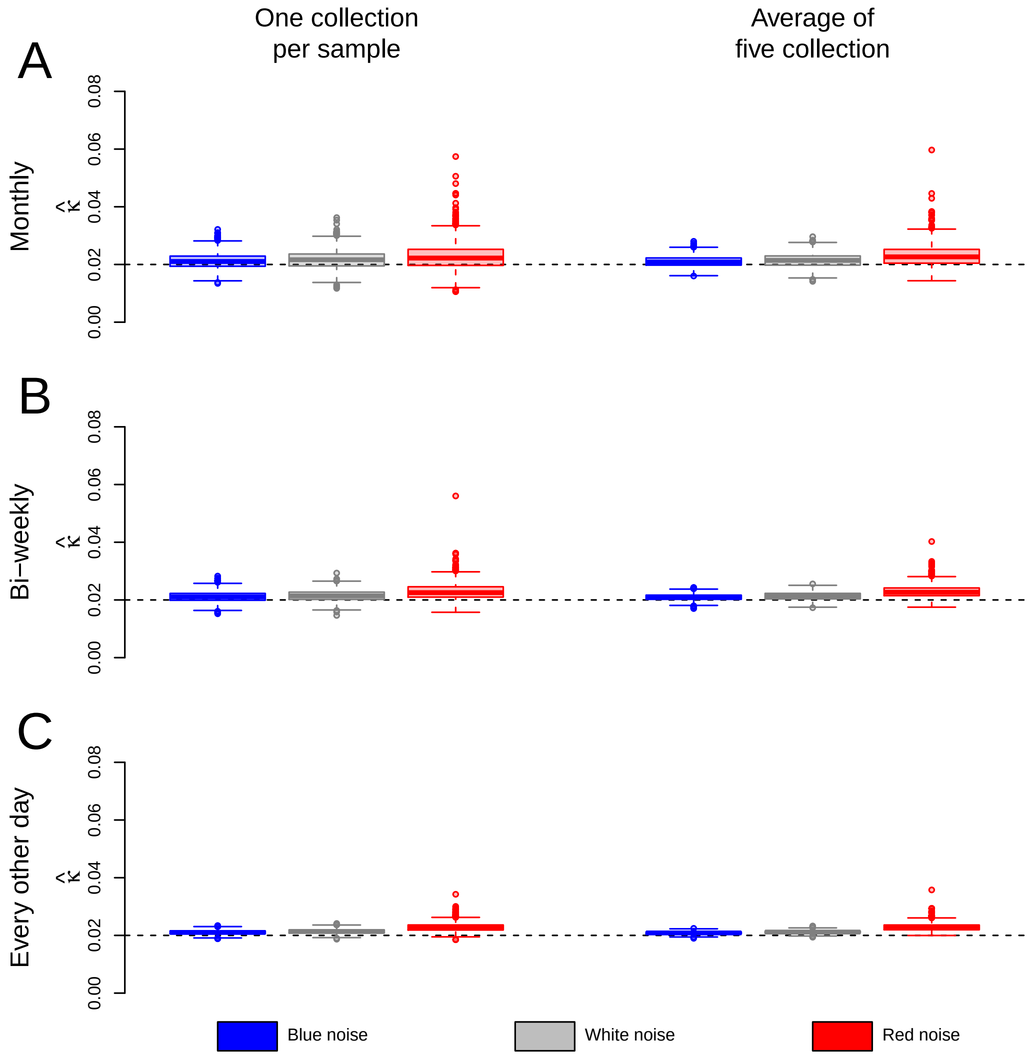

Fig. S6: **Estimating κ with $\hat{\kappa}_3$.** Estimates of κ using the 3rd estimation method are plotted for simulations where emergence incorporated either blue, white and red noise and collections were done every month (panel A), bi-weekly (panel B) or every other day (panel C). Sampling error is assessed by contrasting estimates based on a single collection (left box plots) versus the average of five collections all done on the same night (right box plots). True value indicated by black dashed line.

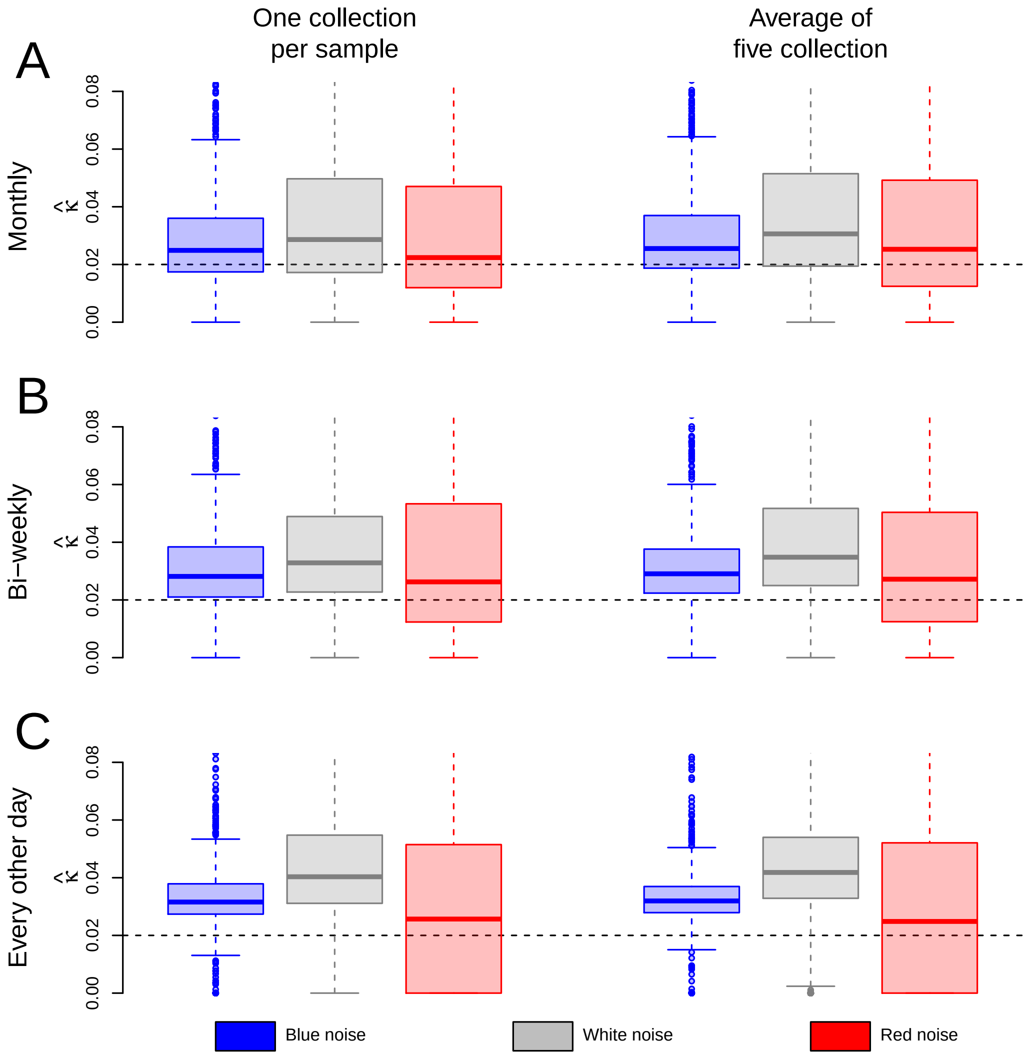

Fig. S7: **Estimating κ with $\hat{\kappa}_4$.** Estimates of κ using the 4th estimation method are plotted for simulations where emergence incorporated either blue, white and red noise and collections were done every month (panel A), bi-weekly (panel B) or every other day (panel C). Sampling error is assessed by contrasting estimates based on a single collection (left box plots) versus the average of five collections all done on the same night (right box plots). True value indicated by black dashed line.

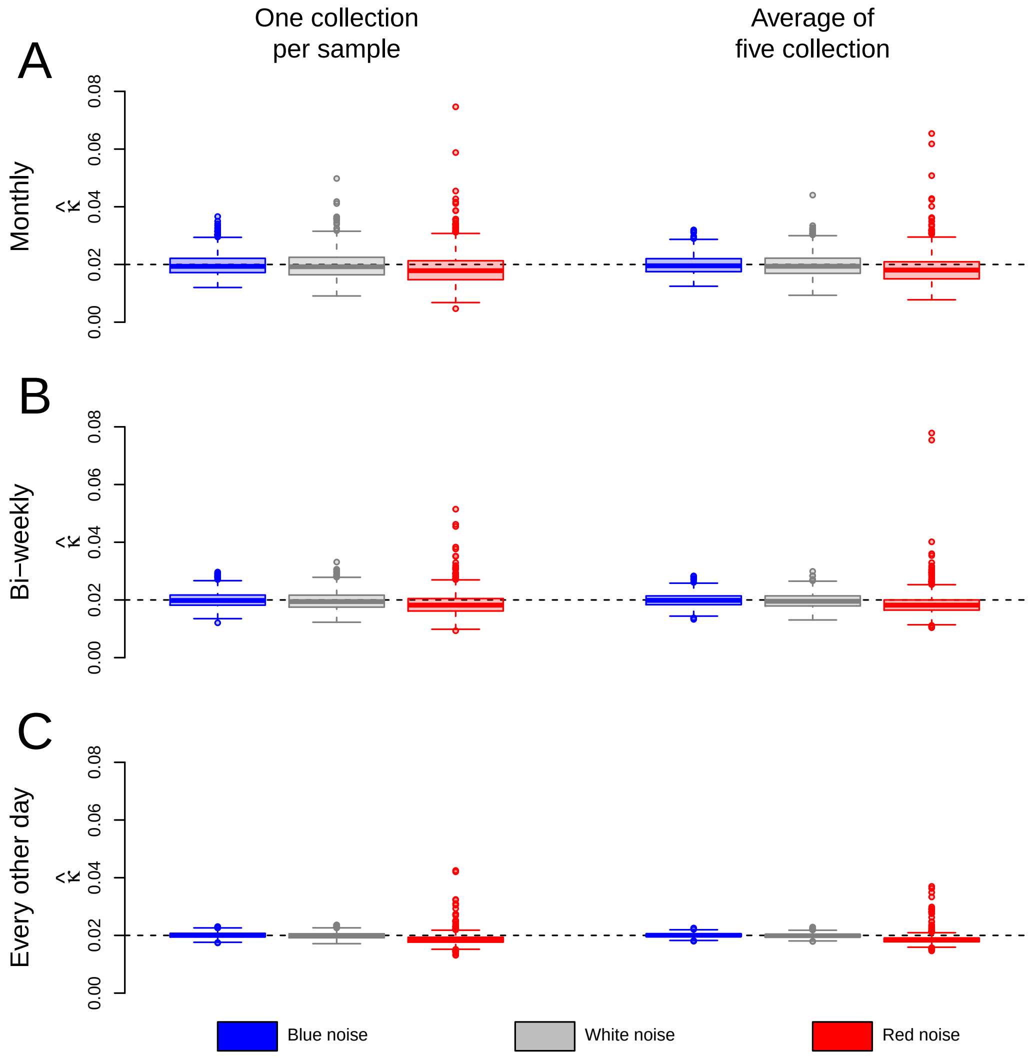

Fig. S8: **Estimating κ with $\hat{\kappa}_5$.** Estimates of κ using the 5th estimation method are plotted for simulations where emergence incorporated either blue, white and red noise and collections were done every month (panel A), bi-weekly (panel B) or every other day (panel C). Sampling error is assessed by contrasting estimates based on a single collection (left box plots) versus the average of five collections all done on the same night (right box plots). True value indicated by black dashed line.

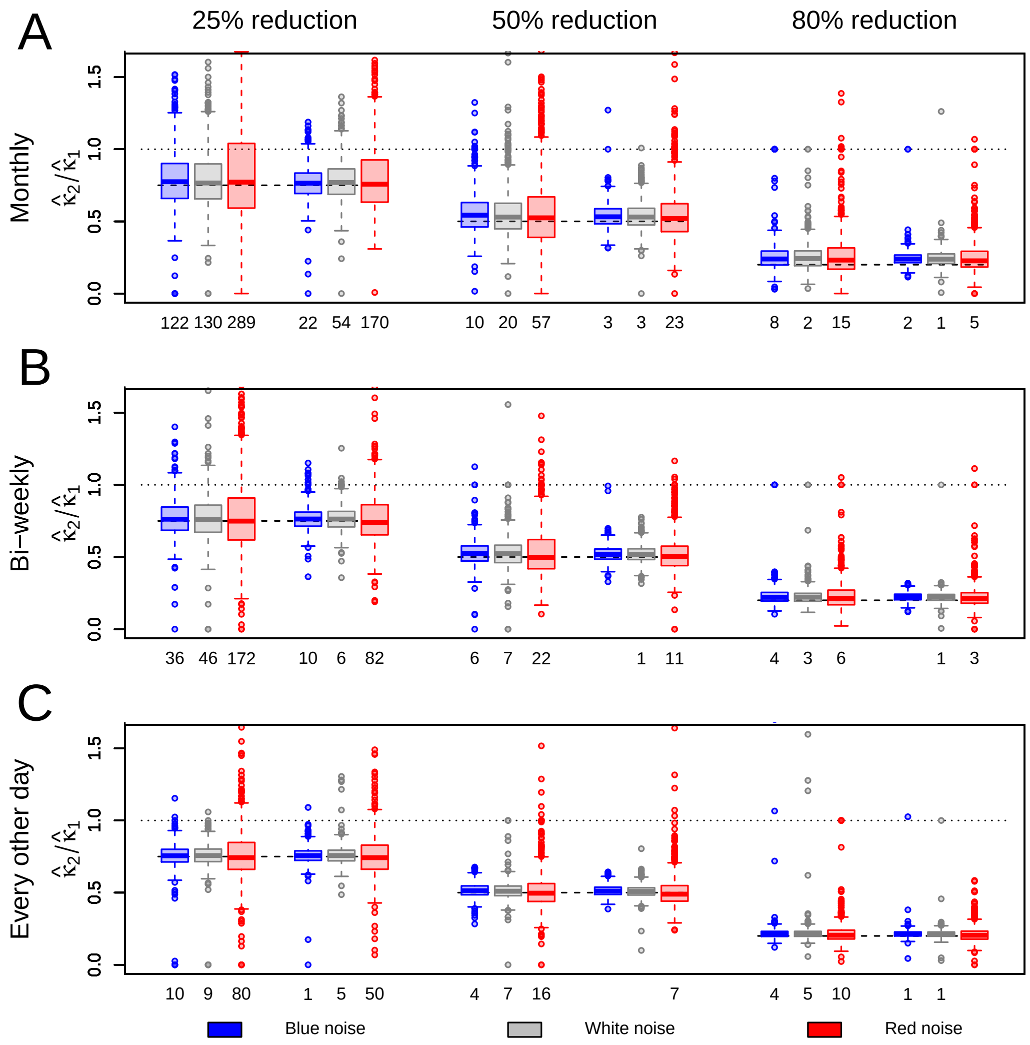

Fig. S9: **Estimating reduction in κ with $\hat{\kappa}_1$.** Reduction in transmission intensity using the 1st estimation method was simulated for three scenarios, 25% reduction (left plots), 50% reduction (middle plots) and 80% reduction (right plots). For each scenario, estimates are plotted for simulations where emergence incorporated blue, white, or red noise and collections were done every month panel A), bi-weekly (panel B) or every other day (panel C). Sampling error is assessed by contrasting estimates based on a single collection (left box plots) versus the average of five collections all done on the same night (right box plots). True value indicated by blue dashed line, while 'no difference' (i.e., $\hat{\kappa}_1 = \hat{\kappa}_2$) indicated with black dotted line. Out of 1000 simulations, the number of simulations where no decrease in transmission intesnity was detected is listed below the corresponding box plot.

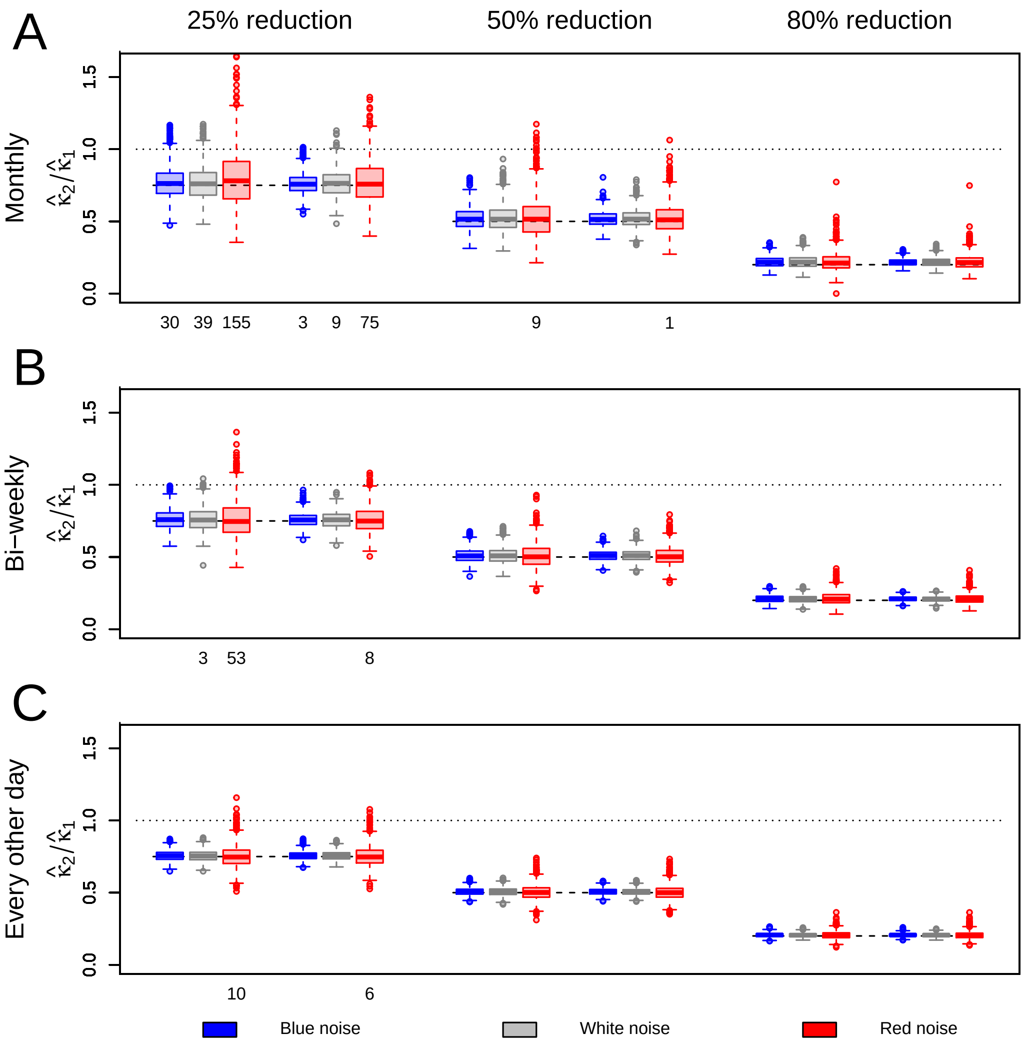

Fig. S10: **Estimating reduction in κ with $\hat{\kappa}_2$.** Reduction in transmission intensity using the 2nd estimation method was simulated for three scenarios, 25% reduction (left plots), 50% reduction (middle plots) and 80% reduction (right plots). For each scenario, estimates are plotted for simulations where emergence incorporated blue, white, or red noise and collections were done every month panel A), bi-weekly (panel B) or every other day (panel C). Sampling error is assessed by contrasting estimates based on a single collection (left box plots) versus the average of five collections all done on the same night (right box plots). True value indicated by blue dashed line, while 'no difference' (i.e., $\hat{\kappa}_1 = \hat{\kappa}_2$) indicated with black dotted line. Out of 1000 simulations, the number of simulations where no decrease in transmission intesnity was detected is listed below the corresponding box plot.

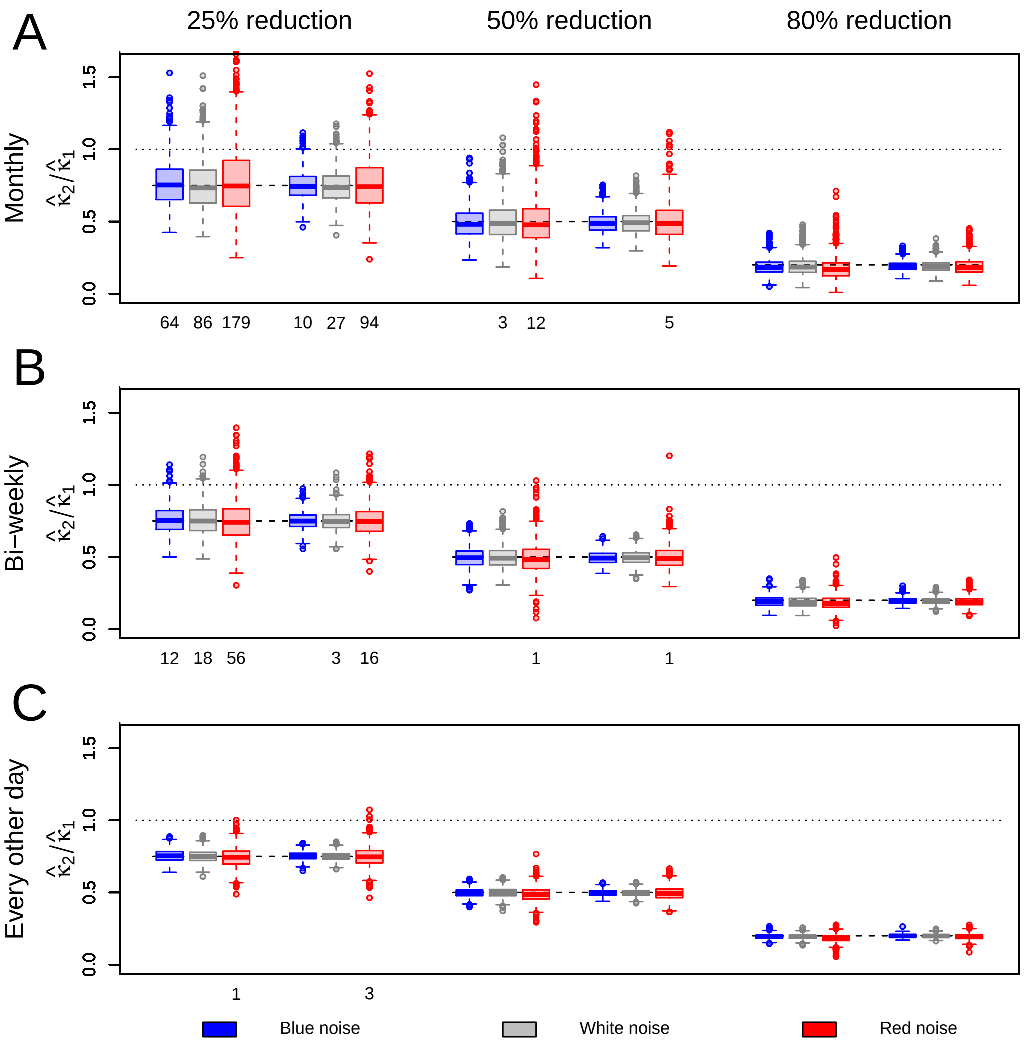

Fig. S11: **Estimating reduction in κ with $\hat{\kappa}_3$.** Reduction in transmission intensity using the 3rd estimation method was simulated for three scenarios, 25% reduction (left plots), 50% reduction (middle plots) and 80% reduction (right plots). For each scenario, estimates are plotted for simulations where emergence incorporated blue, white, or red noise and collections were done every month panel A), bi-weekly (panel B) or every other day (panel C). Sampling error is assessed by contrasting estimates based on a single collection (left box plots) versus the average of five collections all done on the same night (right box plots). True value indicated by blue dashed line, while 'no difference' (i.e., $\hat{\kappa}_1 = \hat{\kappa}_2$) indicated with black dotted line. Out of 1000 simulations, the number of simulations where no decrease in transmission intesnity was detected is listed below the corresponding box plot.

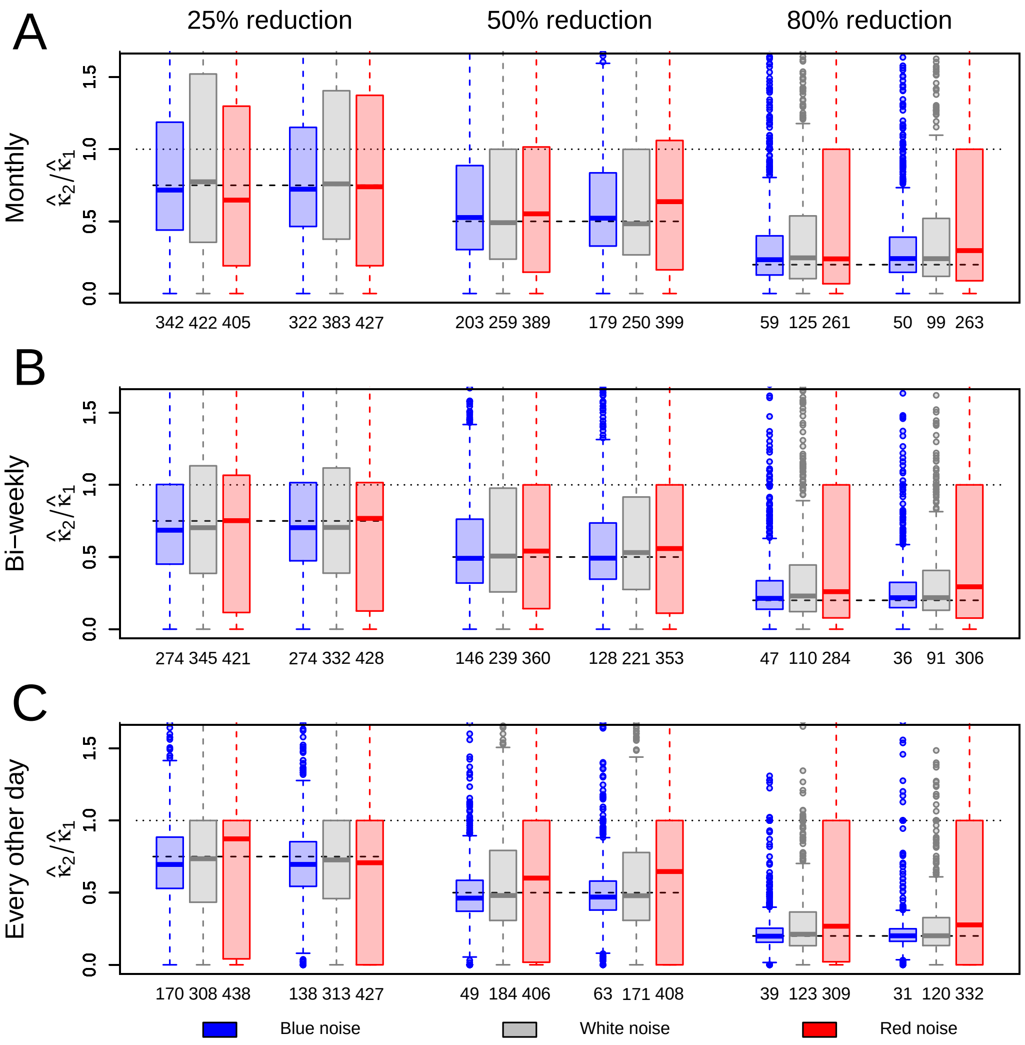

Fig. S12: **Estimating reduction in κ with $\hat{\kappa}_4$.** Reduction in transmission intensity using the 4th estimation method was simulated for three scenarios, 25% reduction (left plots), 50% reduction (middle plots) and 80% reduction (right plots). For each scenario, estimates are plotted for simulations where emergence incorporated blue, white, or red noise and collections were done every month panel A), bi-weekly (panel B) or every other day (panel C). Sampling error is assessed by contrasting estimates based on a single collection (left box plots) versus the average of five collections all done on the same night (right box plots). True value indicated by blue dashed line, while 'no difference' (i.e., $\hat{\kappa}_1 = \hat{\kappa}_2$) indicated with black dotted line. Out of 1000 simulations, the number of simulations where no decrease in transmission intesnity was detected is listed below the corresponding box plot.

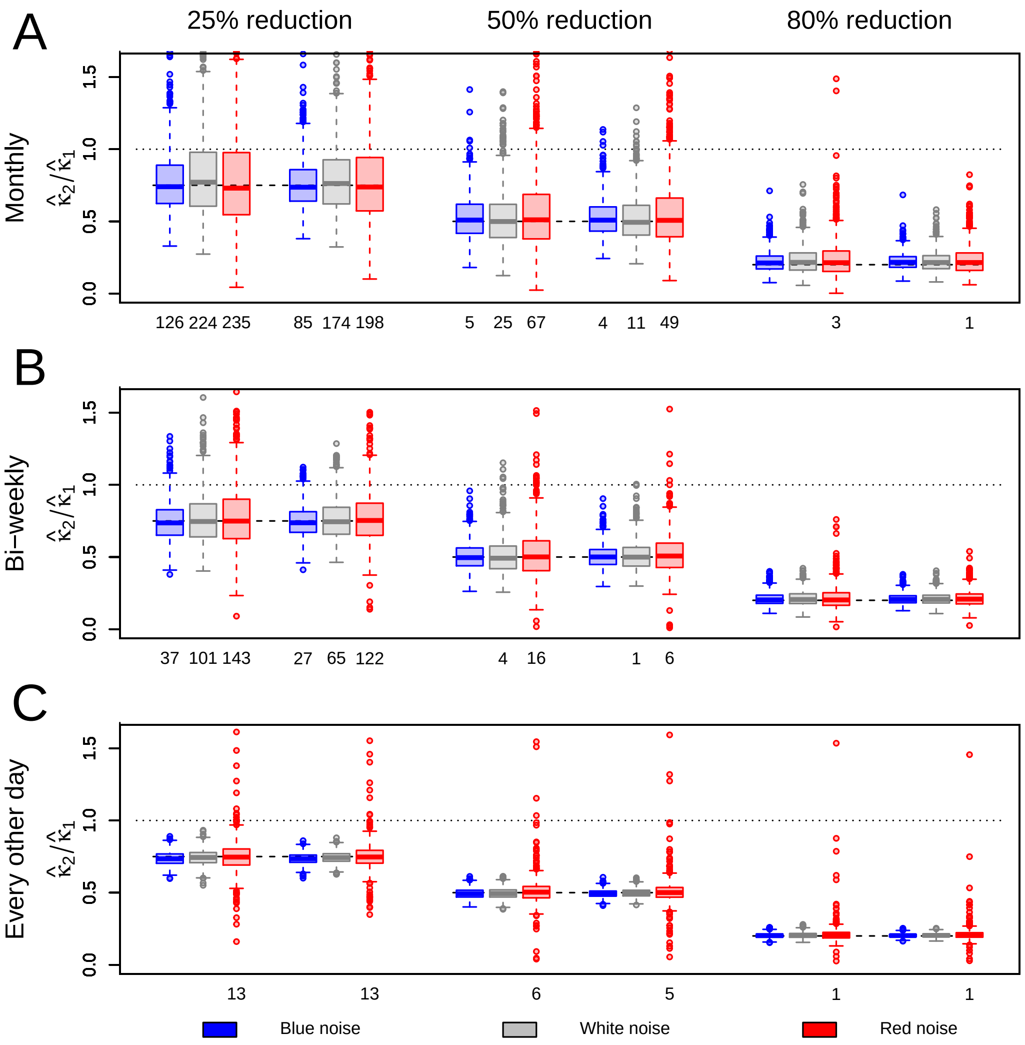

Fig. S13: **Estimating reduction in κ with $\hat{\kappa}_5$.** Reduction in transmission intensity using the 5th estimation method was simulated for three scenarios, 25% reduction (left plots), 50% reduction (middle plots) and 80% reduction (right plots). For each scenario, estimates are plotted for simulations where emergence incorporated blue, white, or red noise and collections were done every month panel A), bi-weekly (panel B) or every other day (panel C). Sampling error is assessed by contrasting estimates based on a single collection (left box plots) versus the average of five collections all done on the same night (right box plots). True value indicated by blue dashed line, while 'no difference' (i.e., $\hat{\kappa}_1 = \hat{\kappa}_2$) indicated with black dotted line. Out of 1000 simulations, the number of simulations where no decrease in transmission intesnity was detected is listed below the corresponding box plot.

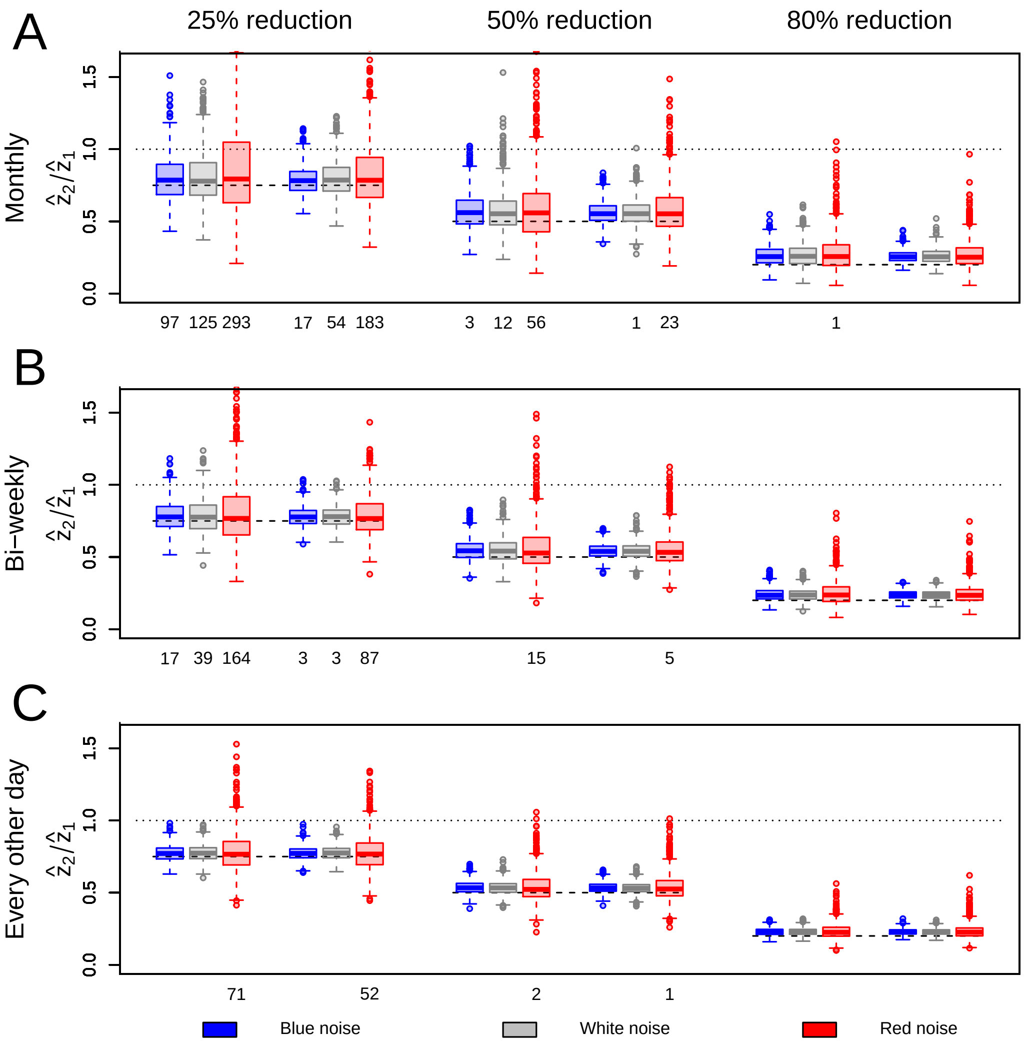

Fig. S14: **Estimating reduction in sporozoite rate.** Reduction in sporozoite rate is estimated for three scenarios, 25% reduction (left plots), 50% reduction (middle plots) and 80% reduction (right plots). For each scenario, estimates are plotted for simulations where emergence incorporated blue, white, or red noise and collections were done every month panel A), bi-weekly (panel B) or every other day (panel C). Sampling error is assessed by contrasting estimates based on a single collection (left box plots) versus the average of five collections all done on the same night (right box plots). True value indicated by blue dashed line, while 'no difference' indicated with black dotted line. Out of 1000 simulations, the number of simulations where no decrease in sporozoite rate was detected is listed below the corresponding box plot.

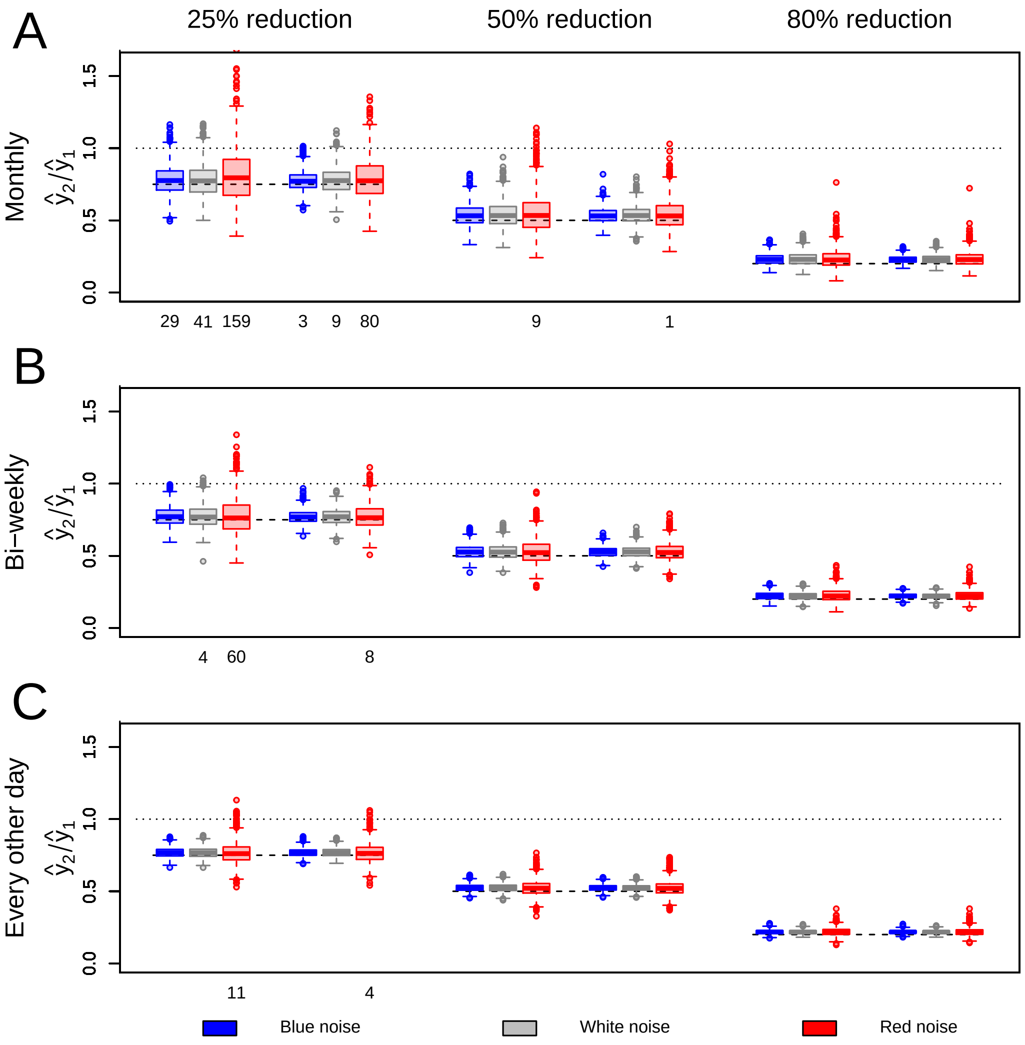

Fig. S15: **Estimating reduction in oocyst rate.** Reduction in oocyst rate is estimated for three scenarios, 25% reduction (left plots), 50% reduction (middle plots) and 80% reduction (right plots). For each scenario, estimates are plotted for simulations where emergence incorporated blue, white, or red noise and collections were done every month panel A), bi-weekly (panel B) or every other day (panel C). Sampling error is assessed by contrasting estimates based on a single collection (left box plots) versus the average of five collections all done on the same night (right box plots). True value indicated by blue dashed line, while 'no difference' indicated with black dotted line. Out of 1000 simulations, the number of simulations where no decrease in oocyst rate was detected is listed below the corresponding box plot.
